# Personality matters in extremely demanding environments: A bed rest performance study

**DOI:** 10.3389/fpsyg.2024.1444276

**Published:** 2024-11-04

**Authors:** Panja Goerke, Claudia Marggraf-Micheel, Dirk Stelling, Henning Soll

**Affiliations:** Department of Aviation and Space Psychology, Institute of Aerospace Medicine, German Aerospace Center, Hamburg, Germany

**Keywords:** extremely demanding environments, bed rest, personality profile, performance criteria, emotional instability

## Abstract

**Introduction:**

Personality is a rather neglected aspect in bed rest studies. The aim of the study was to clarify which specific personality pattern may predict the performance of bed rest study participants.

**Materials and methods:**

Personality traits were correlated with participants’ performance rated by the team running the study. The sample consisted of *N* = 68 participants who took part in one of four different studies. A broad set of personality traits correlated with different performance aspects (stability, perseverance, modesty, flexibility, compliance, likability, social adaptation).

**Results:**

Emotional instability showed the highest correlations. Furthermore, participants with low aggressiveness, low empathy and low achievement motivation were rated as more suitable for a study. Additionally, participants with a high extraversion showed a higher social adaptation.

**Discussion:**

The results contribute to the knowledge of the impact of personality in extremely demanding environments and provide first evidence for the identification of an ideal personality profile predicting performance of bed rest study participants.

## 1 Introduction

Bed rest studies were established during the development of space travel as a measure to simulate weightlessness and the related physiological processes. It became evident that lying in bed for long periods of time represents the closest approximation to inactivity and minimization of hydrostatic effects, both characteristics for living in outer space. Already in the 1970s, it was shown that bed rest combined with 6 degree head-down tilt (HDT) is particularly well suited for research into weightlessness (Pavy-Le Traon et al., 2007). Since then, physiological and psychological effects similar to those observed during a stay in space have been studied in bed rest under HDT for various questions (Pavy-Le Traon et al., 2007; Seaton et al., 2009; Poritz et al., 2012). Concrete issues of HDT research were bone loss, muscle atrophy, cardiovascular changes and sensorimotor alterations (Cromwell et al., 2018). Research on the psychological state of astronauts focused on stress reactions and the development of neurotic behavior through the specific conditions of a space mission. In order to attenuate the strains of space flight, different countermeasures were investigated in HDT studies. For example, Hargens and Vico (2016) reported on measures to reduce bone resorption. Stavrou et al. (2015) examined the influence of extremely demanding environments in terms of bed rest and normobaric hypoxia on the emotional state. They showed that the combined effect of hypoxia and bed rest induced the most negative effects on the individual’s mood and concluded that habitual levels of physical activity might counteract these negative effects. Furthermore, interventions to reduce or even prevent depression and other psychological complaints were investigated (Ishizaki et al., 2002; Ishizaki et al., 2004; Nicolas and Weiss, 2009). Another branch of research focused on changes in cognitive performance during of head-down tilt (e.g., Basner et al., 2018; Basner et al., 2021; Tays et al., 2022; Yuan et al., 2016). Barkaszi et al. (2022) summarize in their review, that most studies found no clear performance impairment during and after HDT exposure. Furthermore, they state that results of HDT studies are not always showing parallel results to space flight, which they try to simulate.

Focusing on bed rest environments, possible environmental stressors are a lack of privacy, a dependency on others and limited social contacts reflecting similar stressors to those encountered during space missions and Antarctic stays (Bischop, 2004; Nicolas et al., 2015; Nicolas et al., 2019). While psychological aspects and the impact of extremely demanding environments on participants’ emotional state have been of research interest, the influence of personality characteristics in bed rest conditions has been rather neglected so far.

Although personality characteristics were not primarily in research focus within adverse environments, there are some empirical hints from isolated and confined environmental conditions suggesting that certain personality traits are important for the well-being of participants and the success of the different missions. A literature search revealed that study results are available on three main personality models, which are presented in the following:

A common classification used for the description of an ideal personality profile of astronauts, pilots and inhabitants of Antarctic environments is the “right stuff.” The “right stuff” includes high levels of positive instrumentality (defined as overall goal seeking and achievement orientation) and positive expressivity (defined as interpersonal warmth and sensitivity) as well as low levels of negative instrumentality (such as arrogance and hostility) and verbal aggressiveness as the negative aspects of expressivity (Chidester et al., 1991; Helmreich and Wilhelm, 1991; Musson et al., 2004b). Research focusing on this personality profile showed some links to performance: Astronauts and pilots with the “right stuff” profile showed higher ratings on social compatibility, training effectiveness and professional effectiveness (Chidester et al., 1991; McFadden et al., 1994; Rose et al., 1994). Furthermore, participants of polar missions and isolation studies with a high positive expressivity and a high positive instrumentality could adapt better to these demanding environments (Sandal et al., 1996).

Another prominent personality model which is widely used to predict performance in non-clinical as well as in work and organizational settings is the “Big Five” personality model. In the last decades, aerospace psychology also increasingly used this model for questions regarding personality in isolated and confined environments (Palinkas et al., 2000; Bischop, 2004; Musson et al., 2004b; Mittelstädt et al., 2016b). The Big Five consist of neuroticism (or the opposite: emotional stability), extraversion, openness to experience, agreeableness and conscientiousness. Especially neuroticism is considered as an essential personality trait in the context of isolation and confinement. Several researchers identified low neuroticism as a central characteristic for an ideal profile in different extreme environmental situations (Manzey et al., 1995; Steel et al., 1997; Palinkas et al., 2000; Musson et al., 2004b; Palinkas and Suedfeld, 2008). Participants with a low level of neuroticism were more powerful and felt more comfortable in isolation during Antarctic stays (Palinkas et al., 2000) and in long duration space flights (Rose et al., 1994; Kanas et al., 2009). Research on extraversion shows ambiguous findings for different occupations and environments. Musson et al. (2004b) observed that astronaut applicants had substantially higher scores on extraversion than students¸ but there are no studies on the relationship to performance. Similarly, Steel et al. (1997) found that circumpolar sojourners scored higher on extraversion than the mean population norm group. However, Palinkas and Suedfeld (2008) described “introverted but socially adapted” and “not greatly extraverted” as ideal characteristics for polar expeditioners. Furthermore, low extraversion was identified as a predictor for high performance in isolated and confined environments (Palinkas et al., 2000; Rosnet et al., 2000). Similarly, research on conscientiousness revealed indistinct results. In comparison to a normative sample of students, astronauts as well as Antarctic personnel had higher scores on conscientiousness. In addition, an intergroup comparison showed that astronauts scored systematically higher on conscientiousness than Antarctic personnel (Musson et al., 2004a). Somewhat contradictory results are reported by Palinkas et al. (2000), who found that low conscientiousness was a significant predictor for high performance in Antarctic environments. In the context of bed rest studies, Seaton et al. (2009) stated that well-organized subjects, who are most likely to have a high value in conscientiousness, may struggle to adapt to changing organizational study circumstances. Reinforcing this it can be stated that even in highly structured bed rest studies, deviations (from the schedule) occur and require a certain flexibility from the participants. This may be an indication that low or medium conscientiousness is rather beneficial for the performance of bed rest study participants. Agreeableness was a positive predictor for different effectiveness dimensions including peer and supervisor ratings of astronauts (Rose et al., 1994). Polar expeditioners scored higher than norm groups on agreeableness (Steel et al., 1997) and Suedfeld and Steel (2000) classify agreeableness as an optimal indicator of adaptive personality functioning. Seaton et al. (2009) highlight the ability to get along with others as an important requirement for a well and successful participation in head-down bed rest studies. No specific research results are known so far concerning the influence of openness to experience – the fifth Big Five dimension – in isolated and confined environmental conditions.

A third model which is used to examine personality in the context of aerospace and aviation research and personnel selection are the Temperament Structure Scales (TSS). The TSS were developed by the Department of Aerospace Psychology at DLR and have their roots in the 1970s. The requirement was to establish a personality inventory which measures relevant variables for aviation and space personnel, robust to social desirability. As a result of its construction under selection conditions, it showed a good reliability in this context and had advantages over other questionnaires (Goeters et al., 1993). The TSS have been successfully used for the selection of aviation personnel and astronauts (Hörmann and Maschke, 1996; Maschke et al., 2011; Pecena et al., 2013; Mittelstädt et al., 2016b) and for the selection of bed rest study participants (blinded for review). The original version consists of 11 scales: achievement motivation, emotional instability, rigidity, extraversion, aggressiveness, vitality, dominance, empathy, spoiltness, mobility and openness (control scale, which is intended to measure social desirability). The TSS showed a considerable relation to the NEO-PI-R (Costa and McCrae, 1992) and can be embedded in the Big Five framework (Mittelstädt et al., 2016a): analyzing two different samples of astronauts and pilot applicants, Mittelstädt et al. (2016a) found out that several TSS scales showed a large overlap with the Big Five. A clear allocation was found for the TSS scales emotional instability (factor neuroticism), extraversion (factor extraversion), achievement motivation and rigidity (factor conscientiousness) in both samples. Other TSS scales like aggressiveness and empathy showed a less clear assignment with loadings on different Big Five factors.

As mentioned before the TSS have the advantage of enabling a differentiated measurement of traits like achievement motivation, aggressiveness, rigidity and empathy, which are relevant for aviation and space personnel but are rather underrepresented in the other common personality profiles. Nevertheless, results in the field of space flights and polar missions stress the importance of these traits. For example, high levels of achievement motivation were consistently found in the population of astronauts (Rose et al., 1994; Musson, 2003; Musson et al., 2004a). For Antarctic and for isolated confinement environments, achievement motivation seems to be less relevant (Suedfeld and Steel, 2000; Musson et al., 2004b). Palinkas et al. (2000) found a negative correlation between conscientiousness and performance criteria and discuss that high conscientious and motivated winter-over personnel might experience depressive symptoms and other performance losses due to the absence of opportunities to meet social and psychological needs. Likewise, extreme achievement motivation might be detrimental for participants of bed rest studies, because these environments are often little controllable, which might frustrate highly motivated participants. Furthermore, the trait empathy shows proximity to positive expressivity (in terms of interpersonal warmth and sensitivity) of the “right stuff” described by Chidester et al. (1991) and Helmreich and Wilhelm (1991) and might therefore be an important personality trait in the field of extremely demanding environments. Sandal et al. (1996) found out, that participants of polar and isolation studies with a high positive expressivity and a high positive instrumentality adapted better to demanding environments.

Concerning the relation between TSS and performance criteria there are only few findings so far. Analyzing the performance of airline pilots, Hörmann and Maschke (1996) could show that successful pilots scored significantly lower on emotional instability, aggressiveness and empathy, which can be subsumed as the emotional scales of the TSS. Furthermore, successful pilots had higher scores on the interpersonal scales extraversion and vitality.

In summary, findings from the different models indicate that people working in extremely demanding environmental conditions share certain similarities differentiating them from other groups. However, findings in one group cannot simply be transferred to another group (Musson et al., 2004a), e.g., because of methodical and situational differences, which might have an influence on motivational aspects and cognitive functions of participants (Barkaszi et al., 2022). Nevertheless, it is often done due to the lack of research (Mittelstädt et al., 2016b). Thus, in this study, it is examined, to which extent findings from other extremely demanding environments about the relevance of personality traits on performance can be transferred to bed rest studies.

Bed rest studies are cost and time intensive and a premature termination of a study results in a total loss of scientific data. They differ from other isolation conditions in their requirements for participants. Only in these environments, participants do not have a regular work assignment and are immobile most of the time. Furthermore, even though they are geographically isolated from their familiar environments, they have enough time to interact with family and friends by social media. Hence, Stavrou et al. (2015) concluded that bed rest studies are intended to examine the effects of immobility, but that they are not a high-fidelity analogy of a space mission simulating the activities of the astronauts. Therefore, research results from other extremely demanding environments cannot be directly transferred to bed rest studies and it is necessary to identify specific performance criteria suitable for the setting of bed rest studies. In this case performance is not related to the measurement of cognitive abilities but to adaptability and social behavior in the study.

A closer look at the overview on personality shows that only few empirical findings for the impact of personality traits on isolation conditions in bed rest studies exist. Moreover, there are not only few empirical findings, but even fewer studies that focus on the performance of the subjects with external criteria. Most studies focus on emotional states like the stability of moods or psychological complaints (e.g., Styf et al., 2001; Ishizaki et al., 2002; Nicolas, 2009). Poritz et al. (2012) stressed the importance of systematically measuring subjects’ performance in the psychologically demanding conditions of bed rest studies. They identified three categories of relevant behavioral performance aspects, namely compliance with rules, interpersonal skills and motivation orientation, and recommended to examine the relationship between psychological screening data and subjects’ performance in future research.

Besides behavioral performance aspects, including different aspects of interpersonal skills and compliance (Poritz et al., 2012), stability was confirmed as an important factor for bed rest study performance (Ishizaki et al., 2002; Nicolas, 2009; Seaton et al., 2009). In addition, based on our study experience, we further identified some additional issues which are relevant for performance in bed rest studies: Participants have to deal with immobility and have to accept loss of control for a longer period of time, which is represented by a high level of perseverance. Furthermore, in the area of interpersonal skills, participants have to be on good terms with care staff and with other participants in order to create a pleasant social climate in the study and facilitate the whole implementation of the study. Thereby, the ability to adapt to other persons (social adaptation) and a friendly and open way of approaching others (likability) has been of advantage for social interaction. Normally, bed rest studies are characterized by routines and protocols. But often enough routines and fixed schedules have to be changed. Therefore, a high flexibility in dealing with changing situations helps to succeed in bed rest. Reinforcing Poritz et al. (2012), we experienced that for scientific control reasons it is very important that participants in bed rest studies show a high level of compliance and that advice from care staff or scientists is followed. Finally, participants who are very demanding or who are extremely depending on social climate are often difficult to handle, which is a problem for care stuff, especially if time or resources are short. In line with these considerations, we experienced that subjects with a high modesty are much easier in daily dealing for the study team.

This study tried to shed light on the question, which specific pattern of personality traits can predict the performance of bed rest study participants. To identify these personality patterns, the relationships to the performance criteria in bed rest studies identified above were investigated: stability, perseverance, compliance, flexibility, social adaptation, likability and modesty. In order to make use of the mentioned advantages of the TSS and at the same time to link to former results of isolation research and personality research, the TSS was considered in the context of the Big Five and the “right stuff” personality model discussed earlier.

Based on findings in all areas of research focusing on isolation environments, we had the following hypotheses:

Emotional instability and aggressiveness (facets of neuroticism) are the most important personality aspects in bed rest study performance. Consequently, we expected:

*H1*: Emotionally stable and little aggressive participants are psychologically robust (stability), have a more positive social interaction (social adaptation, likability, modesty) and deal better with changing and demanding situations (flexibility, perseverance).

Research on extraversion showed ambiguous findings in isolation environments. However, since extraverted people are more sociable, we expected:

*H2*: Higher extraversion makes it easier to adapt to the subject group and the study team (social adaptation).

Based on findings regarding positive expressivity we expected:

*H3*: Higher interpersonal sensitivity (empathy) is positively correlated with social adaptation and with ratings of likability.

Subjects have to deal with immobility and have to accept a loss of control for a longer period of time. People high in achievement motivation get frustrated when they feel or even experience that they have no personal control over a situation (Winter, 2010). Participants with a higher level of achievement motivation are generally active, busy and action oriented. Thus, they might have bigger problems when they are immobile, have to be passive and dependent on other people. Therefore, we expected:

*H4*: Participants with a high achievement motivation find it harder to get through the whole study and have a lower perseverance.

As discussed above, bed rest studies might require a high flexibility of participants when dealing with changing situations in unexpected circumstances. Rigid participants for whom it is not easy to adapt to changing situations are likely to be bothered by this. Thus, we expected:

*H5*: Participants, who are very principle minded and who are not flexible (rigidity) are rated as less adaptable to changing situations (flexibility) in bed rest studies.

## 2 Materials and methods

Since 2001, bed rest studies have been carried out at DLR on behalf of organizations such as NASA and ESA or by DLR itself. Standards for HDT bedrest studies (Cromwell et al., 2018) were considered when conducting the studies in specially equipped research facilities.

The data presented here is based on four separate bed rest studies: (1) MEP (Medium-Term Bed Rest Whey Protein) study with 2 × 21 days of HDT bed rest; (2) RSL (Reactive Jumps in a Sledge Jump System) study using jumps as a countermeasure with 60 days of HDT bed rest; (3) VaPER [VIIP (Visual Impairment and Intracranial Pressure) and Psychological: envihab Research] study with 30days of HDT bed rest; (4) AGBRESA (Artificial Gravity Bed Rest with European Space Agency) study with 60 days of HDT bed rest conducted by DLR. Different countermeasures were used in the above-mentioned studies: MEP focused on a dietetic factor, the RSL study used horizontal jumps in a sledge system, the VaPER study examined visual disorders and cerebral pressure and the AGBRESA study investigated artificial gravity (AG) exposure. The experimental protocol and the implementation of each of the four studies including duration and experimental conditions are described in Buehlmeier et al. (2014, MEP), Kramer et al. (2017, RSL), Clément et al. (2022a, VaPER) as well as in Clément et al. (2022b, AGBRESA).

Each of the four bed rest studies had the same structure with three phases: 14 days baseline, a bed rest phase with a length of 21, 30 or 60 days, respectively, and a recovery phase of 14 days. The baseline phase was used for familiarization with the daily routine of the study, the recovery phase for muscle building and restoring overall fitness. Experiments were conducted in all study phases.

During the bed rest phase, subjects were restricted to a 6-degree HDT position. Throughout the entire period of the study, all participants were housed in single rooms and they could use a bathroom with a shower daily (while lying down). They received standard meals three times each day. During the whole study, physicians checked the physical condition of the subjects. The participants received full support from the study team to cope with everyday life. Contact to visitors from outside was not allowed during the study, but the participants could use the telephone or internet to stay in touch with their family and friends or for professional concerns. In leisure times, the internet, books and films were available. In addition, contact to other study participants could be established via social media and (video) calls and also directly in the common room – all during the bed rest phase while lying down.

The study protocols for each of the four bed rest studies were approved in advance by the Ethics Committee of the Medical Council of North Rhine (Ärztekammer Nordrhein) in Düsseldorf, Germany: MEP (application No. 2010426/11–151), RSL (application No. 2014105) VaPER (application No. 2016408), AGBRESA (application No. 2018143). Each subject was informed about the research objectives, risks, key experiments and research methods and provided written consent before participating.

### 2.1 Procedure

All applicants of the four bed rest studies took part in a multi-stage selection process and were thoroughly selected based on medical and psychological criteria. Each of the four studies had its own selection process. The medical criteria for each of the studies are described in Buehlmeier et al. (2014, MEP), Kramer et al. (2017, RSL), Clément et al. (2022a, VaPER) as well as in Clément et al. (2022b, AGBRESA). The psychological fit was assessed by testing the applicants in terms of compliance, resilience, motivation and social skills to determine their suitability (Poritz et al., 2012). The whole selection process was administered by DLR. A pre-selection including a personality questionnaire and a biographical questionnaire was followed by a medical screening and a semi-structured interview in the last selection stage.

Subjects’ study performance in terms of psychological fit and subjects’ behavior during all phases were assessed by the study team at the end of the study via questionnaire. Thereby, assessors were instructed to provide their evaluation regarding the entire study period.

### 2.2 Subjects

In total 400 candidates applied to be part of one of the four bed rest studies (MEP, RSL, VaPER, AGBRESA) and completed the first selection stage including a psychological pre-screening (personality and biographical questionnaire) and a medical screening. Only 151 applicants reached the second selection stage and participated in an interview and a further final medical exam. The ultimate study sample consisted of 68 healthy subjects (55 men, 13 women; age: *M* = 31.8, *SD* = 7.6 years) belonging to the four bed rest studies: The MEP study included 10 male subjects (age: *M* = 32.7, *SD* = 5.3 years). The RSL study consisted of 23 male subjects (age: *M* = 29.5, *SD* = 6.0 years) and was conducted within two campaigns (campaign 1: *N* = 12, campaign 2: *N* = 11; one drop-out due to medical reasons in campaign 2). The VaPER study examined 6 male and 5 female subjects (age: *M* = 32.8, *SD* = 8.1 years) within one campaign (one female dropout due to psychological reasons), and the AGBRESA study included 16 male and 8 female subjects (age: *M* = 33.13, *SD* = 9.25 years) within two campaigns of 12 subjects each. An *a priori* power analysis conducted using G*Power (Faul et al., 2009) determined a sample size of N = 67 to be sufficient for detecting a medium effect size (*r* = 0.30) with a power of 0.8.

### 2.3 Measures

#### 2.3.1 Temperament Structure Scales (TSS)

For this study, we used the German version, developed for applicants without any specific experience in aviation. It consists of 180 items (15 per scale and 30 items for the scale openness) and a forced two–choice (yes/no) answering format for given statements or choosing one of two alternatives for self-descriptions. Raw scores were standardized using a stanine scale. Descriptions of relevant personality dimensions are presented in Table 1. The TSS scale for Openness measures social desirability and self-presentation and should not be confused with the Big Five factor of Openness to experience (Goeters et al., 1993; Stelling, 2023). Specific scales relevant for the selection of student pilots only (like mobility, vitality) were not evaluated in this study.

**Table 1:** Description of low and high scores in the Temperament Structure Scales (TSS).

TSS Scales	Low score	High score
Emotional instability	Resilient, optimistic	Nervousness, easily frustrated
Aggressiveness	Peaceable, diplomatic	Impulsive, obstinate
Extraversion	Reserved, does not mind being alone	Sociable, lively
Empathy	Rational, hard-hearted	Sympathetic, altruistic
Achievement motivation	Avoids effort, enjoys life	Ambitious, always busy
Rigidity	Spontaneous, no sense of order	Tactical, principle minded
Openness	Denies own weakness, always ideal behavior	Admits weakness, admits non-conformist behavior

The TSS had no time limit and applicants were required to answer all items. Cronbach’s Alpha for each of the 10 original scales ranged from *α* = 0.61 to *α* = 0.87 (mean *α* = 0.83) in an *ab initio* pilot candidate sample (Goeters et al., 1993) and from *α* = 0.69 to *α* = 0.89 in a more recent sample of astronauts and *ab initio* pilot candidates (Mittelstädt et al., 2016b).

#### 2.3.2 Subjects’ study performance

Based on literature research (Poritz et al., 2012) and on our experience, seven important aspects of study performance were identified (see Supplementary Table 1):

1. Stability (STA) – item 1, item 2
2. Perseverance (PER) – item 3
3. Modesty (MOD) – item 4
4. Flexibility (FLE) – item 5
5. Compliance (CPL) – item 6, item 7
6. Likability (LIK) – item 8, item 9, item 10
7. Social adaptation (SAD) – item 11, item 12

Subjects’ study performance was rated via questionnaire by the study team consisting of study nurses, scientists, kitchen staff, study lead, physicians, and others. The sample of raters varied between the studies and the different study campaigns, respectively (MEP: *N* = 29, RSL: *N*_campaign 1_ = 18, *N*_campaign 2_ = 14, VaPER: *N* = 20, AGBRESA: *N*_campaign 1_ = 32, *N*_campaign 2_ = 31). The assessors were instructed to rate the participants’ study fit and behavior based on their observations during the whole study. Ratings were given on a four-point Likert scale ranging from 1 to 4 (for example 1 = not suitable to 4 = suitable). No significant differences were found between the ratings given by the different professional groups (e.g., study nurses, scientists, kitchen staff) of the study team. The inter-rater agreement ICC (2, k) ranged between 0.70 and 0.99. Therefore, mean scores per scale across all raters were calculated.

### 2.4 Statistical analysis

All data was stored in a SQL database and all statistical analyses were conducted using SPSS 21 software^1^. In order to analyze the factor structure of the TSS scales and their relation to the Big Five dimensions, a factor analysis was conducted. Using principal component analysis with a varimax rotation, it was examined, whether the used TSS scales reflected the corresponding Big Five dimensions. The Kaiser–Guttman criterion was chosen as cut-off, including only factors with eigenvalues greater than one. For stable results, the total sample of applicants attending an interview (*N* = 151) was included in this analysis.

Pearson correlation coefficients were used to assess the relationships of the TSS scales with the performance criteria for the final study sample (*N = 68.*. Relationships between personality and performance variables might be confounded by social desirability. Therefore, partial correlations were used to examine the relationship between personality and performance. Computing these partial correlations, the influence of the TSS scale openness on the relationship between personality and performance was controlled.

## 3 Results

Descriptive statistics for personality scales and performance criteria in the final study sample (*N = 68.* can be found in Table 2.

**Table 2:** Descriptives for personality and performance criteria.

	*M*	*SD*	Minimum	Maximum
Personality				
Emotional instability	4.69	2.09	1	9
Aggressiveness	4.26	1.79	1	9
Extraversion	5.10	2.21	1	9
Empathy	5.84	1.88	2	9
Achievement motivation	4.69	1.81	2	9
Rigidity	5.21	2.04	2	9
Openness	4.88	1.92	1	9
Performance Criteria				
Stability	3.26	0.60	1.48	3.93
Perseverance	3.57	0.51	1.25	4
Modesty	3.25	0.66	1.50	4
Flexibility	3.38	0.58	1.55	4
Compliance	3.55	0.50	1.42	4
Likability	3.51	0.42	1.88	3.98
Social adaptation	3.31	0.55	1.56	3.97

*N = 68.*

Analyzing the data structure, we have checked spreading and outliers and decided to use Pearson correlation for statistical analysis. Intercorrelations of personality scales ranged between *r* = 0.03 (*ns*) (*Aggressiveness* and *Achievement motivation*) and *r* = 0.35 (*p* < 0.01) (*Achievement motivation* and *Empathy*). The control scale *Openness* showed highly significant correlations with almost all other TSS scales [*Emotional instability r* = 0.54 (*p* < 0.001); *Aggressiveness r* = 0.43 (*p* < 0.001); *Extraversion r* = −0.26 (*p* < 0.05); *Empathy r* = −0.29 (*p* < 0.05); *Achievement motivation r* = 0.01 (*ns*); *Rigidity r* = −0.37 (*p* < 0.01)], indicating the need for partial correlations in order to analyze the correlations between personality dimensions and performance criteria.

Performance criteria showed high significant intercorrelations ranging between *r* = 0.69 (*p* < 0.001) and *r* = 0.92 (*p* < 0.001).

The results of the principle component analysis with a subsequent varimax rotation for the total sample of applicants (*N* = 151) are shown in Table 3. Factor columns are sorted by eigenvalue. The first factor is characterized by high loadings of the TSS scales *Emotional instability* and *Aggressiveness* and therefore corresponds to the neuroticism dimension of the Big Five personality model. The second factor represents the Big Five dimension extraversion with high loadings of the TSS scales *Extraversion* and *Empathy*. Finally, the third factor is dominated by high loadings of the TSS scales *Rigidity* and *Achievement motivation* and can thus be classified as the Big Five dimension conscientiousness.

**Table 3:** Principal component analysis with varimax rotation of TSS scales.

	Factors			
	1	2	3	*h* ^2^
Emotional instability	**0.86**			0.76
Aggressiveness	**0.71**		−0.40	0.69
Extraversion	−0.32	**0.81**		0.76
Empathy		**0.76**		0.63
Achievement motivation	0.34	0.44	**0.54**	0.60
Rigidity			**0.85**	0.76

*N = 151. Loadings below 0.3 are omitted. Bold indicates the highest loading of a scale on a factor. h2 = commonality of each scale.*

The partial correlation matrix of personality dimensions with the performance criteria is presented in Table 4. Confidence intervals for correlation coefficients are listed in Supplementary Table 2.

**Table 4:** Partial correlation matrix of personality and performance criteria.

	STA	PER	MOD	FLE	CPL	LIK	SAD
Emotional instability	−0.26^*^	−0.29^*^	−0.18	−0.30^*^	−0.31^*^	−0.32^*^	−0.29^*^
Aggressiveness	−0.26^*^	−0.20	−0.17	−0.26^*^	−0.25^*^	−0.22^†^	−0.16
Extraversion	0.10	0.15	0.12	0.19	−0.04	0.09	0.30^*^
Empathy	−0.29^*^	−0.23^†^	−0.30^*^	−0.28^*^	−0.21^†^	−0.26^*^	−0.21^†^
Achievement motivation	−0.28^*^	−0.25^*^	−0.22^†^	−0.23^†^	−0.21^†^	−0.22^†^	−0.10
Rigidity	−0.07	0.02	−0.13	−0.06	0.00	−0.00	−0.07

*N = 68.* **p < 0.01; *p < 0.05; ^†^*p* < 0.10 (two-tailed). STA, stability; PER, perseverance; MOD, modesty; FLE, flexibility; CPL, compliance; LIK, likability; SAD = social adaptation.

In detail, the evaluation revealed that *Emotional instability* was correlated significantly negative with most of the performance criteria indicating that emotionally stable participants tended to perform better than emotionally instable participants (H1). Similarly, *Aggressiveness* as a second aspect of neuroticism was highly negatively correlated with stability, flexibility, compliance as well as, marginally, with likability (H1). This indicates that subjects with low *Aggressiveness* scores were attributed with a better performance than participants with high *Aggressiveness* scores. As expected, *Extraversion* was only significantly positively related with social adaptation (H2). In contrast to our hypothesis, *Empathy* was significantly negative correlated with most of the performance criteria (H3). Concerning the personality cluster conscientiousness, the TSS scale *Achievement motivation* was significantly negatively correlated with several performance criteria (H4). Highest negative correlations between *Achievement motivation* and performance criteria were found with stability and perseverance, whereas *Achievement motivation* was not significantly correlated with social adaptation. The TSS scale *Rigidity* was not correlated at all with any of the performance criteria (H5).

## 4 Discussion

The aim of the study was to clarify which specific pattern of personality traits can predict the performance of bed rest study participants. Relevant TSS scales for bed rest studies were identified by a literature review and were embedded in the framework of the “right stuff” and the Big Five personality model in order to establish a connection to previous research in this field. To answer the research questions, the TSS personality traits were considered with regard to different bed rest specific aspects of performance allowing to assess behavioral outcomes of participants.

Facets of neuroticism were expected to be the most important personality aspects for bed rest study performance (H1). Reinforcing our hypothesis, the results of our analysis show that higher scores in *Emotional instability* were significantly correlated with lower ratings in all performance variables. Modesty is the only performance variable that did not show a significant correlation with *Emotional instability*. Our results illustrate, that *Emotional instability* is not only associated with mood, but has an impact on other bed rest specific performance criteria like perseverance, likability, compliance and social adaptation. For *Aggressiveness*, defined in the TSS as the tendency to react in an impulsive and obstinate way toward other people, similar relations to performance criteria were observed. Negative correlations were detected between high scores of *Aggressiveness* and all performance variables and became significant for stability, flexibility and compliance. Both variables scored on the same factor, usually called neuroticism. Thus, our results are in accordance with the findings of other studies (Rose et al., 1994; Palinkas et al., 2000; Nicolas, 2009), which identified neuroticism as one of the most important personality traits for extremely demanding environments like spaceflights and Antarctic missions. For bed rest studies, Nicolas (2009) has shown that a low level of neuroticism is beneficial for handling stress factors. Furthermore, Seaton et al. (2009) emphasized emotional stability as an essential criterion to select participants for bed rest studies. Underlining these aforementioned findings, Ishizaki et al. (2002) have additionally proven that neurotic levels even increased during bed rest. Thus, our result stress the importance of emotional stability in future selection procedures not only for spaceflights but also for bed rest studies.

In accordance with our second hypothesis (H2), extraverted participants were rated higher in social adaptation to other participants and to care staff. We did not find significant correlations with other performance criteria, but our data showed that this personality trait is important whenever there is social contact. These results expand the findings of earlier research carried out in the context of other isolated and confined environments that found a negative relation of extraversion with performance criteria (Palinkas et al., 2000; Rosnet et al., 2000). Whereas they focused on cognitive performance (Rosnet et al., 2000) and identified low extraversion as a significant predictor for general performance measured by peer nominations of ideal winter-over candidates (Palinkas et al., 2000), we could show that *Extraversion* has a positive influence in terms of social oriented performance. Nevertheless, it has to be mentioned that even though results in our analysis indicated a positive relation of extraversion with social oriented aspects of performance in bed rest studies, extremely high values of extraversion might be disadvantageous. Subjects with extremely high values of extraversion require much more attention and would probably have more problems with being dependent on others due to their isolated and immobile position.

We expected (H3) that participants with higher *Empathy* were rated higher in their social fit (social adaptation, likability). Contrary to our hypothesis, high *Empathy* was correlated negatively with all study performance variables at least at the 10% significance level. These negative correlations were unforeseen, especially in the context of likability. However, this becomes more plausible when considering that low *Empathy* may be associated with rational thinking and handling situations in a reasonable way. At the same time *Empathy* was the only personality variable that correlated significantly negatively with modesty. This might reflect that participants with high interpersonal sensitivity are more demanding for care staff, which might have influenced the ratings of all other performance variables as well. Probably, participants with a higher interpersonal sensitivity interpreted behavior of the care team more emotionally and sometimes reacted in an inappropriate manner. In addition, participants’ coping mechanisms for social stressors could be more emotionally driven, which make these participants more demanding for a study team. In the field of aviation, Hörmann and Maschke (1996) could show a link between low empathy and high job performance. Bearing in mind that low empathy can be associated with a rational and rather down to earth behavior in social situations this could be another explanation making the negative correlations more plausible.

We expected that participants with a high motivation for achievement found it harder to get through the whole study time because of a permanent feeling of “loss of control” (H4). According to our hypothesis, the analyses showed a significant negative correlation between high *Achievement motivation* and perseverance. Participants with high *Achievement motivation* were also rated as less stable in moods (stability). This might reflect the impact of feeling “loss of control” on mood.

Finally, we expected that participants with a high *Rigidity* would have more difficulties to adapt to changing situations in bed rest studies (H5). Not supporting our hypothesis, we did not find any correlations between *Rigidity* and flexibility. On the one hand, bed rest studies are characterized by fixed protocols and routines, which might be convenient for principal minded participants. On the other hand, changes in routines might be uncomfortable for very rigid participants. We did not have control about how often and how strong divergences occurred in our study. Therefore, reactions on changing routines might be subject for further research in these kinds of studies.

Taken together, this study confirmed that some of the relationships between personality and performance criteria found in other isolated and confined environments can be transferred to the field of bed rest studies. Especially *Emotional instability* seems to be a crucial personality dimension. Furthermore, low *Empathy* in terms of rational thinking and less emotional reactions as well as a low *Aggressiveness* represent important personality dimensions in this special context. Higher *Extraversion* as well as a lower *Achievement motivation* appear to be less important, but still relevant, in the context of social fit. However, these results show that the ideal personality profile of bed rest participants differs from the personality profile required for spaceflights. This reinforces considerations of Barkaszi et al. (2022) and Musson et al. (2004a), stating that results from one group in extremely demanding environments cannot be transferred directly to another group due to differences in the environment.

As reported, only few studies in extremely demanding environmental conditions evaluated subject performance in the past, let alone having used the same performance measurements. Expanding former bed rest research, to our knowledge, this study is the first to point out relationships of personality with behavioral performance criteria measured by an external source (study team). Thus, the presented relationships have a special weight as we used external criteria for verification.

The results of this study provide an orientation for future bed rest studies’ subject selection. The identified personality traits should be considered during a selection process. Furthermore, our results can be used to detect possible problems due to personality traits already before the start of a bed rest study. On this basis, the study staff can prepare itself to deal with difficulties that might occur. Thereby, the team would be poised to counteract problems in an efficient way and, eventually, the subjects could be supported, encouraged and carried through difficult phases. As a limitation of our study, it has to be mentioned that two factors of the Big Five personality model, namely agreeableness and openness to experience, were not examined. Furthermore, we only focused on some of the TSS facets that were previously identified as relevant for bed rest study performance. However, it might be useful to identify other facets of personality traits and see how they interact with diverse demands in bed rest settings. Thus, in future studies these personality dimensions should be assessed and evaluated.

Another limitation is the pre-selection of participants. Participants with a lack of motivation, a lack of stress resistance, insufficient willingness to adhere to procedures (compliance) and/or a low social fit were selected out. With an unselected sample, correlations would presumably be higher. Unselected samples are a wish from the research point of view, but are unlikely to happen due to the high costs of drop outs.

As a further limitation the unequal distribution of gender in the sample has to be mentioned. Gender might have an influence on the relationship between personality traits and performance criteria. However, the subsample of women was too small, which precluded a calculation of gender differences or an inclusion of gender as a control variable.

In addition, bed rest studies differ in their performance demands: the environment, the need for social interaction, the amount of loss of control or the length of study time are important factors, which might interact with participants’ personality, too. Therefore, a category system for types of bed rest studies for the comparison of psychological research results would be useful.

## 5 Conclusion

Psychological factors in bed rest studies are far less investigated than physiological ones. Our results contribute to the knowledge of the impact of personality in these extremely demanding environments. They provide first evidence for the identification of an ideal personality profile predicting performance of bed rest study participants. In order to successfully complete a bed rest study, participants need to be emotionally stable to deal with the demanding situation of immobility and partial confinement as well as showing a low aggressiveness level concerning the interaction with others. In addition, our results show that a rather rational approach to deal with socially demanding situations (low empathy) is beneficial for bed rest studies. In contrast to other adverse environments like space missions or polar expeditions achievement motivation seems to be less important for bed rest subjects and might be even counterproductive if participants have difficulties to deal with uncontrollable and changing situations in bed rest studies. Taken together, our results emphasize the importance of psychological factors for bed rest studies concerning selection and supporting of subjects, as a drop out would entail high costs.

## Data Availability

The datasets presented in this article are not readily available because the data clearance of the participants only covers the handling by the authors and does not allow any disclosure to third parties. Requests to access the datasets should be directed to Panja Goerke, panja.goerke@dlr.de.

## Footnotes

^1^ https://www.ibm.com/de-de/spss
