# Supplementary material for: Personality matters in extremely demanding environments: A bed rest performance study
Source: Front Psychol. 2024 Nov 4;15:1444276. doi: 10.3389/fpsyg.2024.1444276 (PMC11570997; doi:10.3389/fpsyg.2024.1444276)
Supplement: Supplementary file 1 [file Table_1.pdf]

Table 1 *Performance Criteria items.*

	Items (response scale)	Areas
1	How did the subject stand the pain and was capable of suffering during examinations? (4 = resistant/robust – 1 = sensitive/self-pitying)	Stability (STA)
2	Did the subject suffer from mood swings? (4 = no mood swings – 1 = strong mood swings)	
3	How do you rate the subject's tendency to abort? (4 = no tendency to abort – 1 = strong tendency to abort)	Perseverance (PER)
4	How demanding was the subject regarding your work area? (4 = low demands/expectations – 1 = high demands/expectations)	Modesty (MOD)
5	How did the subject react to changing conditions? (4 = high adaptability/flexibility – 1 = low adaptability/flexibility)	Flexibility (FLE)
6	How suitable was the subject for fulfilling the tasks within your area of the study? (4 = suitable – 1 = not suitable)	Compliance (CPL)
7	How do you rate the subject's compliance? (4 = high compliance – 1 = low compliance)	
8	How friendly/pleasant was the subject during the entire study? (4 = friendly/pleasant – 1 = unfriendly/unpleasant)	Likability (LIK)
9	How sincere/honest was the subject? (4 = sincere/honest – 1 = insincere/dishonest)	
10	How likable was the subject? (4 = likable – 1 = unlikable)	
11	How did the subject adapt to the social structure of the subject group? (4 = high adaptability – 1 = low adaptability)	Social Adaption (SAD)
12	How did the subject adapt to the social structure of the study staff? (4 = high adaptability – 1 = low adaptability)	
