# Supplementary material for: Personality matters in extremely demanding environments: A bed rest performance study
Source: Front Psychol. 2024 Nov 4;15:1444276. doi: 10.3389/fpsyg.2024.1444276 (PMC11570997; doi:10.3389/fpsyg.2024.1444276)
Supplement: Supplementary file 2 [file Table_2.pdf]

Table 2 *95% CI* for *Partial correlation matrix of personality and performance criteria.*

		STA	PER	MOD	FLE	CPL	LIK	SAD
Emotional instability	upper	-.0223	-.0554	.0610	-.0663	-.0772	-.0883	-.0554
	lower	-.4693	-.4942	-.4012	-.5024	-.5106	-.5188	-.4942
Aggressiveness	upper	-.0223	.0404	.0713	-.0223	-.0123	.0194	.0815
	lower	-.4693	-.4185	-.3925	-.4693	-.4609	-.4355	-.3837
Extraversion	upper	.3305	.3750	.3484	.4098	.2003	.3215	.5025
	lower	-.1418	-.0917	-.1219	-.0507	-.2757	-.1517	.0663
Empathy	upper	-.0554	-.0089	-.0063	-.0445	.0299	-.0223	.0299
	lower	-4942	-.4440	-.5024	-.4860	-.4270	-.4693	-.4270
Achievement motivation	upper	-.0445	-.0123	.0194	.0089	.0299	.0194	.1418
	lower	-.4860	-.4609	-.4355	-.4441	-.4270	-.4355	-.3305
Rigidity	upper	.1713	.4185	.1119	.1810	.2384	.2384	.1713
	lower	-.3034	-.0404	-.3573	-.2942	-.2384	-.2384	-.3034

Notes. $N = 68$. STA = stability, PER = perseverance, MOD = modesty, FLE = flexibility, CPL = compliance, LIK = likability, SAD = social adaptation.
